# Quaternary Ammonium Silica Nanoparticles for Antimicrobial Implantable Medical Devices: An In Vitro Study

**DOI:** 10.3390/life14121654

**Published:** 2024-12-12

**Authors:** Eitam Weiss, Ariel Berl, Ofir Shir-az, Biader Samih Bilal, Ervin I. Weiss, Yossi Paitan, Natan Zaltsman, Alexander Golberg, Avshalom Shalom

**Affiliations:** 1 Department of Plastic Surgery, Meir Medical Center, Kfar Saba 4428164, Israel; eitamw@gmail.com (E.W.); arielberl23@gmail.com (A.B.); ofir.shiraz@gmail.com (O.S.-a.); biader.bilal@gmail.com (B.S.B.); 2 School of Medicine, Faculty of Medical and Health Sciences, Tel Aviv University, Tel Aviv 6997801, Israel; 3 School of Dental Medicine, Faculty of Medical and Health Sciences, Tel Aviv University, Tel Aviv 6997801, Israel; ervinw@tauex.tau.ac.il; 4 Clinical Microbiology Laboratory, Meir Medical Center, Kfar Saba 4428164, Israel; yossi.paitan@clalit.org.il; 5 Department of Clinical Microbiology and Immunology, Faculty of Medical and Health Sciences, Tel Aviv University, Tel Aviv 6997801, Israel; 6 Department of Research and Development, Nobio, Ltd., Kadima 6092000, Israel; 7 School of Mechanical Engineering, Faculty of Engineering, Tel Aviv University, Tel Aviv 6997801, Israel

**Keywords:** biofilm, antibacterial technology, antimicrobial QASi particles, nanoparticles, silicone

## Abstract

Biofilm formation on prostheses and implanted devices can lead to serious complications and increased healthcare expenditures. Once formed, biofilm management is difficult and may involve a long course of antibiotics, additional surgery, and, occasionally, implant removal. This study evaluated the antibacterial properties of medical-grade silicone samples integrated with novel, non-leaching, antibacterial, quaternary ammonium silica (QASi) particles. Our collaborators (Nobio, Israel) prepared silicone sheets integrated with antibacterial QASi nanoparticles. Samples containing 0.5%, 0.75%, and 1%, QASi particles were evaluated for antibacterial properties against *S. epidermidis*, *S. aureus*, methicillin-resistant *S. aureus* (MRSA), *E. faecalis*, and *P. aeruginosa* using the direct contact test. The tested silicone samples integrated with QASi particles showed no bacterial growth, while growth was observed in control silicone samples without QASi. In addition, the agar diffusion test, used to evaluate the leaching of antibacterial components, exhibited no inhibition zone around the samples indicating that the QASi particles do not leach into surrounding milieu. The QASi nanoparticles exhibited very potent antibacterial surface properties, killing all viable bacteria placed on their surface. Incorporating QASi nanoparticle technology into medical products during production has the potential to create an antimicrobial surface that prevents microbial colonization and biofilm formation.

## 1. Introduction

Implantable medical devices are susceptible to microbial colonization and biofilm formation, leading to clinical symptoms and device failure. The increased risk of infection is due to the exceptionally resistant nature of biofilms and to the difficulty of the host’s immune system with responding successfully [1]. Once formed, biofilm management is complex and may involve a long course of antibiotics, additional surgery, and, occasionally, implant removal. According to the Centers for Disease Control, over half of healthcare-associated infections are associated with various indwelling medical devices. Mortality rates may reach 25% for certain implanted devices [2,3,4]. *Staphylococcus epidermidis*, *Staphylococcus aureus*, other coagulase-negative staphylococci, and, to a lesser extent, *Propionibacterium* species and *Corynebacterium* are considered among the most prominent pathogens associated with implantable devices [4,5].

Acute infections associated with implantable devices usually occur during the first weeks after surgery and are frequently associated with swelling, fever, pain, erythema, and bacteremia. Late infections are rare and often result from bacteremia or an invasive procedure at a remote location, such as a dental procedure. Once a device is colonized, the body’s immune system struggles to fight and clear all bacteria cells adhering to it [6,7]. In breast reconstruction and esthetic surgery, chronic inflammation from bacteria or biofilm is believed to be associated with the development of capsular contraction and of breast-implant-associated anaplastic large-cell lymphoma, a distressing complication that has caused changes in the regulatory system and the breast implant industry [8,9].

One promising approach for controlling device-associated biofilm infections is to use devices with antibacterial surface properties. An example is leachable antibacterial coatings, such as silver or copper, used in urethral catheters. However, their use is limited because of toxicity, the emergence of bacterial resistance, and impaired host response [10].

The area of nanotechnology is receiving significant interest in the medical field, and the use of this technology to solve problems such as implantable device failures and infections is increasing rapidly [11]. A recent study demonstrated that quaternary ammonium-coated silica (QASi) nanoparticles in resin-based dental composites resulted in a device with long-lasting, potent antimicrobial activity that did not leach into the surroundings. It was also reported that low concentrations of QASi effectively killed saliva microorganisms, consisting of hundreds of diverse species, demonstrating the broad spectrum of antimicrobial activity, including against *Candida* spp. [12,13,14,15].

Dental restorative materials containing QASi have been cleared by the US Food and Drug Administration for use in humans. Furthermore, it was claimed that the antibacterial properties are permanent without decay of the materials, rendering this a novel approach to decreasing bacterial growth and complications that also has a unique safety profile [16,17]. Therefore, quaternary ammonium nanoparticles can potentially be incorporated into various implanted medical devices [18].

This study describes the incorporation of QASi nanoparticles into medical-grade silicone. The antibacterial properties of the modified silicone nanoparticles were tested for their activity against the most common clinical pathogens involved in implantable device infections.

## 2. Materials and Methods

### 2.1. Antimicrobial Particles

**Figure 1:**
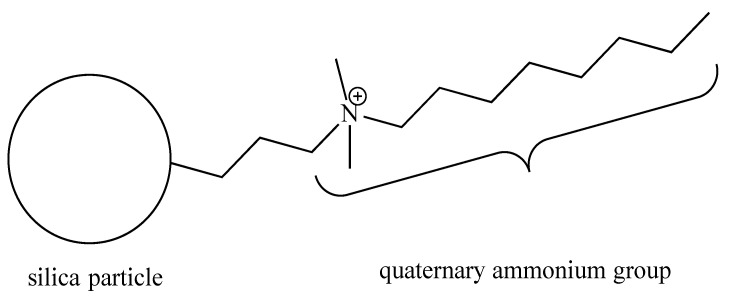
Schematic structure of the quaternary ammonium silica particle (QASi). The quaternary ammonium groups are built of one C-8 alkyl and two C-H_3_ groups attached to a nitrogen cation (N^+)^, which is covalently bonded to an inert silica dioxide solid particle.

The quaternary ammonium silica (QASi) nanoparticles (Figure 1) were prepared by our collaborators (Nobio^TM^, Ltd., Kadima, Israel). The QASi nanoparticles were synthesized to form a high concentration of antimicrobial groups [13] that are covalently bound onto silica, which serves as a carrier core [19]. The resulting micro- or nano-size QASi particles were mixed into medical-grade silicone (Nusil Technology, LLC, Carpinteria, CA, USA) before the silicone polymerization process at the Nobio laboratory.

### 2.2. Materials Tested

**Figure 2:**
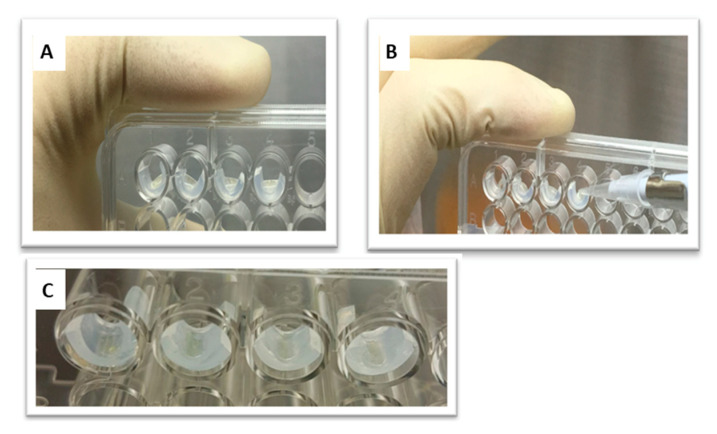
(**A**) A 96-well microtiter plate. (**B**) A total of 10 µL of the bacterial suspension was placed on the tested silicone samples while the plate was held vertically. (**C**) The suspension was dried by evaporation to ensure contact with the silicone samples.

Samples of medical-grade silicone containing concentrations of QASi nanoparticles of 0%, 0.5%, 0.75%, and 1% weight/weight (*w*/*w*) were prepared. The nanoparticles were incorporated into the silicone immediately before the polymerization reaction. Polymerization was performed according to manufacturing protocols. Silicone samples, approximately 3 mm × 6 mm, containing nanoparticles (Figure 2) were prepared to fit the side walls of the wells in a 96-well plate (MicroWell Plates, Nunc, Copenhagen, Denmark).

Briefly, two-part medical-grade silicone rubber monomers MED-6600 (NuSil Technology, LLC, Carpinteria, CA, USA) were mixed for 30 min using an overhead stirrer according to the manufacturer’s instructions. Separately, a dispersion of 30% wt. QASi particles in xylene (Sigma Aldrich, Rehovot, Israel) was prepared using a high-shear homogenizer (Ultraturax T25, IKA, Staufen, Germany). All final QASi concentrations in the silicone samples were calculated, taking into account that the polymer content in the MED-6600 is 60% and the rest are volatile solvents (xylenes). According to these calculations, the amount of QASi in the xylene dispersions was added to the MED-6600 pre-mixed solutions. Consequently, each sample was poured into an aluminum weighting boat, making an approximately 1 mm layer of liquid. The samples were inserted into a ventilated box-type oven, first at 60 °C for 30 min and then at 80 °C for another 30 min to allow for solvent evaporation. The samples were then allowed to polymerize by elevating the oven temperature to 180 °C and leaving it for 2 h. Subsequently, the samples were peeled from the aluminum bottom to be used in this study.

### 2.3. Test Microorganisms

The silicone samples were tested against the following bacterial strains: *S. epidermidis*, *S. aureus*, *MRSA*, *E. faecalis*, and *P. aeruginosa*. These bacteria are commonly associated with infections and failure of silicone-based implantable devices in humans. The bacterial strains used in the present study were obtained from ATCC strains (Manassas, VA, USA) and clinical isolates. The clinical isolates were provided by the Department of Microbiology, Meir Medical Center, Kfar Saba, Israel.

The microorganisms were maintained on agar plates according to their growth requirements. Prior to each experiment, the relevant strain was cultured in 5 mL brain–heart infusion (BHI) broth for 24 h at 37 °C under aerobic conditions. Strains were examined by spectrophotometry to reach an optic density of 0.06–0.1 with a wavelength of 650 nm. This wavelength is equivalent to 1–5 × 10^6^ CFUs (colony-forming units).

### 2.4. Experimental Design

#### 2.4.1. Direct Contact Test (DCT)

The DCT, first described by Weiss et al. [20], is designed to measure the bacteriostatic and bactericidal properties of solids that do not secrete or leach the antibacterial components. Briefly, silicone test samples of each QASi concentration were placed in the wells of a 96-well microtiter plate. A 10 µL inoculum of bacterial suspension (approximately one million viable bacteria, estimated by viable counts) was placed on the surface of each test and control surface in the wells (Figure 2). The 96-well plate was incubated vertically for 1 h at 37 °C to allow for the evaporation of the media liquid and for the bacterial cells to come into contact with the tested solids (Figure 2). Thereafter, 240 µL growth medium (BHI) was added to each well, and the plate was incubated at 37 °C in a temperature-controlled spectrophotometer. Optical absorbance was measured at 650 nm in each well, every 30 min for at least 12 h. iControl software version 1.6.19.2 (iControl Systems USA, LLC, Bethesda, MD, USA) was used to collect data on bacterial growth, which were then transferred to Microsoft Excel (Redmond, WA, USA) for further analysis.

Experiments were performed in 96-well plates, and each plate was an independent experiment; therefore, they included appropriate positive and negative controls. Each plate also included triplicate wells with serially diluted bacterial inoculum to allow for the calculation of absolute numbers of live bacteria per well and to compare data between plates [20].

**Figure 3:**
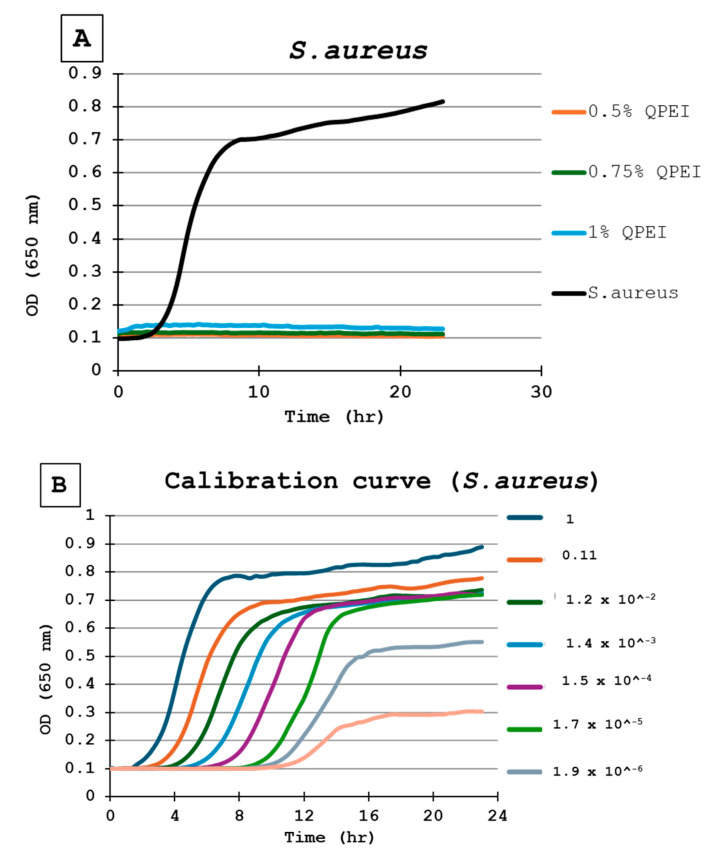
(**A**) Bacterial growth kinetics following direct contact with *S. aureus*. Each point on the curve is the mean absorbance (A650 nm) measured in 8 wells. All were similarly prepared in the same microtiter plate. 0.5% QPEI—orange; 0.75% QPEI—green; 1% QPEI—blue; composite without QASi nanoparticles—black. (**B**) *S. aureus* calibration curves. Ninefold dilutions were performed seven times in triplicate, resulting in 8 growth curves in the same microtiter plate. Each point on the curve is the mean absorbance (A650 nm) measured in 3 wells.

For the calibration of each experiment in each microtiter plate, the same inoculate of bacteria was inoculated in triplicate wells. Then, a ninefold dilution was performed seven times, in triplicate, resulting in numerous growth curves, until no bacteria were present, as indicated by a flat line (Figure 3A). Each point in the growth curve represents the average of 3 wells, independently measured at each time point (Figure 3B).

#### 2.4.2. Agar Diffusion Test

The agar diffusion test (ADT) was used to evaluate antibacterial effects around the silicone test samples, as it is usually used to observe the inhibition zone around antibiotic disks. Test bacteria were suspended in 400 µL BHI and spread over the entire surface of sheep blood agar plates using Sterilin™ Loops and Spreaders (Thermo Fisher Scientific, Inc., Waltham, MA, USA). After the fluid of the suspensions had soaked into the agar, the silicone samples were placed on the agar, along with antibiotic disks (amoxicillin/clavulanic acid (AMC 30), cefuroxime (CXM 30), and ofloxacin (OFX 5)) as positive controls. The plates were incubated at 37 °C for 48 h. When an inhibition zone was observed, the inhibition halo diameter was measured with a caliper in two perpendicular directions. The ADT was performed with silicone samples containing 0.5% and 0.75% QASi nanoparticles and for each of the following bacteria: *S. epidermidis*, *MSSA*, *MRSA*, *E. faecalis*, and *P. aeruginosa*.

#### 2.4.3. Dynamic Light Scattering Characterization of the Silica Nanoparticles

A total of 10 mg of QASi particles was dispersed in 10 mL of Type I water using a high-shear homogenizer (Ultraturax T25, IKA, Staufen, Germany) until a clear dispersion was obtained. The prepared dispersion was then analyzed for the size distribution and the zeta potential of the QASi using a dynamic light scattering device (Malvern Zetasizer, Malvern, UK).

### 2.5. Data Analysis

The absorbance measurements were plotted to provide bacterial growth curves for each well in the microtiter plate. The growth curves for each well were analyzed, and a regression line on the ascending linear portion of the curve was calculated, using the following equation: y = ax + b. Here, the value corresponded to the logarithmic growth phase. The growth curves were created using Microsoft Excel (Microsoft Corp., Redmond, WA, USA). Two-sided *p*-values < 0.05 were considered significant.

## 3. Results

### 3.1. Direct Contact Test

**Figure 4:**
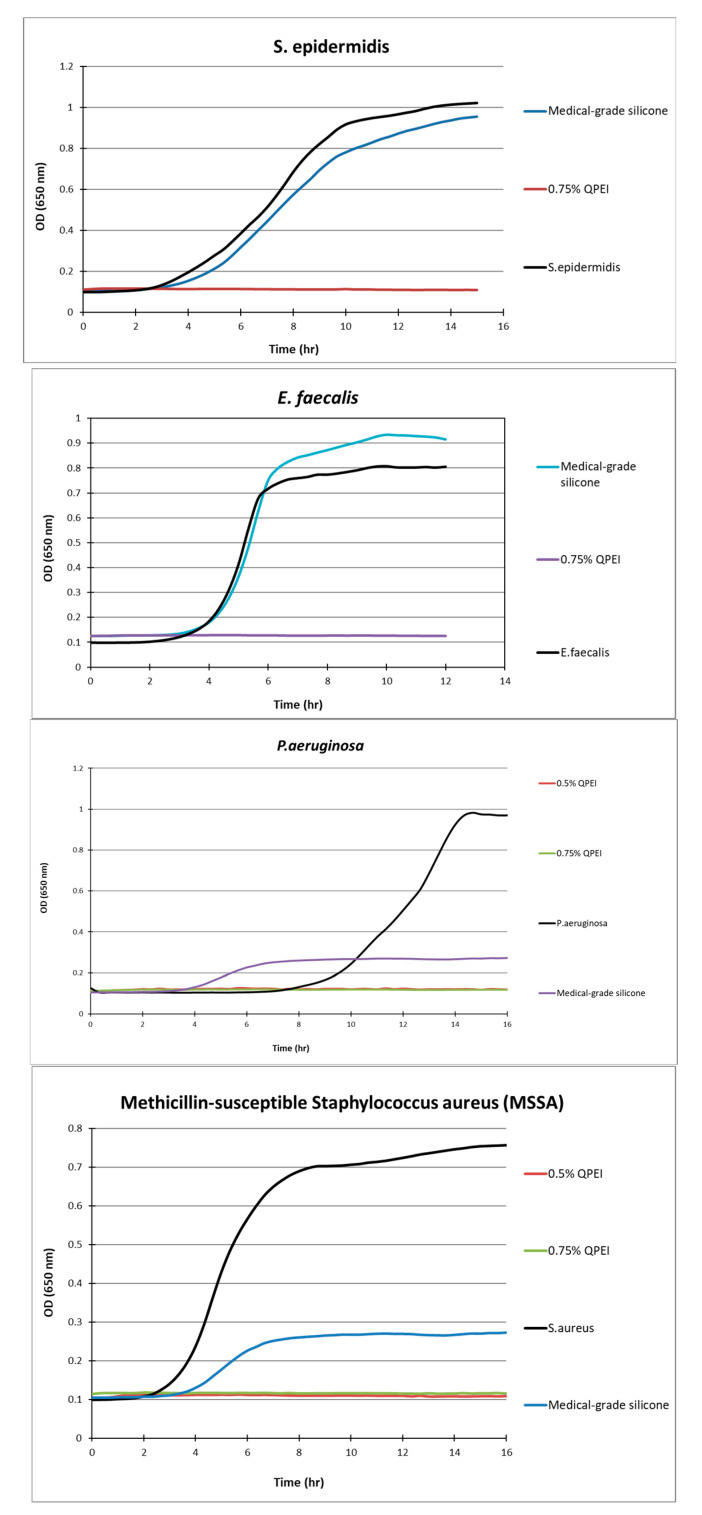
Kinetic measurements of bacterial growth following direct contact between *S. aureus*, *S. epidermidis*, *E. faecalis*, *P. aeruginosa*, and silicone samples with 0.75% *w*/*w* incorporated QASi nanoparticles. The growth of surviving bacteria on the surface was measured every 30 min. Each point on the curve is the average absorbance (650 nm) measured in 8 replica wells, prepared similarly, in the same microtiter plate.

The DCT was performed on silicon specimens with 0%, 0.5%, 0.75%, and 1% concentrations of QASi nanoparticles for each bacterium tested. The calibration curves of the experiment and growth curves of the microorganisms are shown in Figure 3 and Figure 4. The points on each growth curve are the mean values measured in 8 identical wells in the same 96-well microtiter plate. The calibration experiments showed no bacterial growth (straight line) in the last dilution after 11 h. The ninefold dilutions (Figure 3) yielded different calibration growth curves, from which the actual number of viable bacteria at the beginning of the experiment was calculated.

The DCT shows that the composite containing QASi particles inhibited *S. aureus* growth; a straight line in the growth curve after 24 h of incubation indicates no bacterial growth (Figure 3A). Based on the calibration curves (Figure 3B), at least 1 million live cells were inoculated. Thus, even if one single bacterium survived, it would start to grow 8–10 h after the beginning of the experiment. In contrast, at least 106 viable bacteria grew on the surface of the same formulation control composite without the QASi particles and on the plastic (polystyrene) surface (Figure 3A).

After more than 12 h of incubation, no bacterial growth was measured in the experimental wells containing silicone samples in which 0.5% or 0.75% of QASi nanoparticles were incorporated. Thus, *S. epidermidis*, *E. faecalis*, *P. aeruginosa*, and methicillin-sensitive *S. aureus* did not grow on the silicone surfaces containing QASi. However, on the control silicone samples, typical bacterial growth curves were measured, comparable to the inert control surfaces of the wells (polystyrene surface).

### 3.2. Agar Diffusion Test

**Figure 5:**
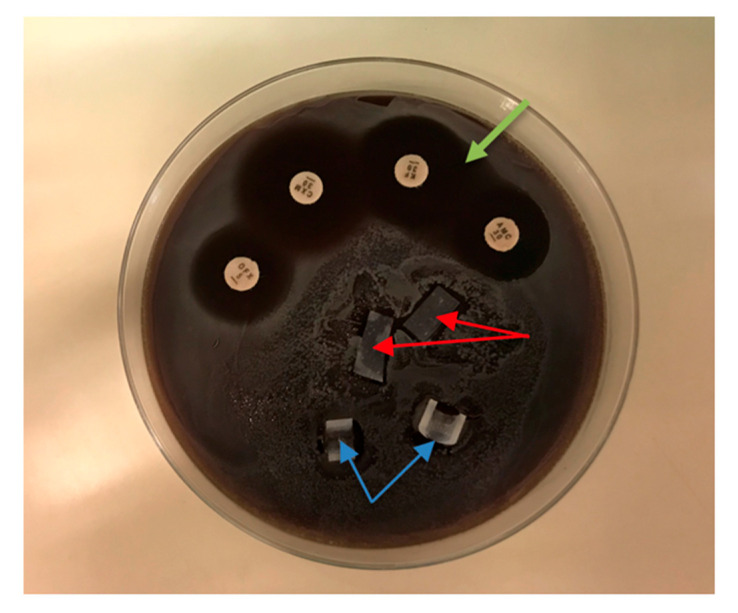
Agar diffusion test of *S. aureus*. Silicone samples containing 0.5% nanoparticles (red arrows) and samples containing 0.75% (blue arrows) showed no bacterial growth inhibition. Inhibition zones (green arrow) were obtained around the antibiotic disks: amoxicillin/clavulanic acid (AMC 30), cefuroxime (CXM 30), and ofloxacin (OFX 5).

In the ADT, inhibitory halos of 5.0 ± 0.3 mm were found around the amoxicillin/clavulanic acid, cefuroxime, and ofloxacin disks after 48 h of incubation. There was no inhibitory halo around the silicone samples containing 0.5% and 0.75% nanoparticles, indicating virtually no leakage of antibacterial components into the surrounding area (Figure 5). Similar results were obtained for the other microorganisms tested (*S. epidermidis*, *MSSA*, *MRSA*, *E. faecalis*, and *P. aeruginosa*).

### 3.3. Dynamic Light Scattering

Size distribution: QASi particles size measurements were collected and calculated as an average of three runs, showing that the average size was 147 nm, with the peak at 187 nm and a standard deviation of 110 nm. The polydispersity index of 0.243 indicates good uniformity of particles and a stable sample.

**Figure 6:**
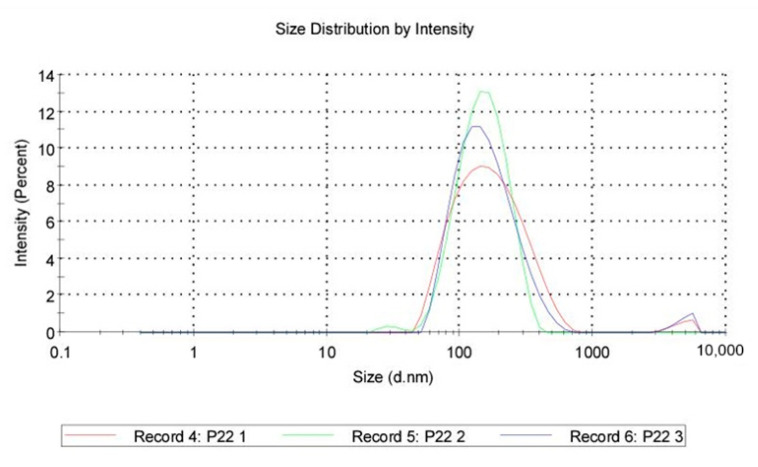
Quaternary ammonium silica particle (QASi) size distribution curves.

**Figure 7:**
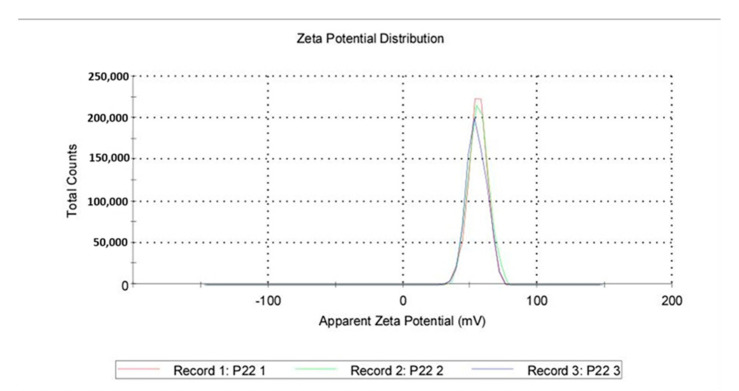
Quaternary ammonium silica particle (QASi) zeta potential curves.

Zeta Potential: A total of 1 mL from a previous dispersion was examined for the zeta potential induced by the QASi particles, and the results are represented as an average of three runs. The average zeta was 55.5 mV, with a standard deviation of 6.96 mV (Figure 6 and Figure 7).

## 4. Discussion

Contamination of implanted medical devices during surgery or through late bacteremia could result in an infection that may lead to device failure and negative outcomes. The customary practice is to treat the infection empirically with broad-spectrum antibiotics while awaiting culture results to avoid complications and morbidity due to the infection and, potentially, device removal. Various methods for preventing bacterial contamination when inserting an implantable device have been described. For silicone breast implants, this includes chlorhexidine skin preparation, which entails soaking the implant in a triple antibiotic solution and irrigating the implant pocket before insertion using a no-touch technique, along with using prophylactic antibiotics in the perioperative period. Other treatment and prevention strategies to prevent biofilm formation on indwelling catheters and implantable devices have been described and introduced to the medical field. These are mainly preventative, aimed at reducing bacterial inoculum and growth, and include maintaining sterile techniques, along with technologies incorporated into the devices to reduce bacterial inoculum and colonization [21].

A different group of strategies, such as polymer coatings aimed at changing the physiochemical properties of the surface along with a bactericidal effect, has been described [22,23,24,25]. However, their efficacy in vivo declines over time. Implantable devices, such as catheters, that are permeated with antimicrobial material are available, but their effect on catheter-associated infections has been questioned [26]. As shown in previous studies, bacterial biofilm formation depends on cell adhesion and proliferation, and the nano-specific topography of surfaces can affect and possibly control these properties [21,27,28,29].

Beyth et al. described incorporating quaternary ammonium cross-linked polyethyleneimine (QPEI)-based nanoparticles into dental resin composites, resulting in a potent antibacterial effect against *Streptococcus mutans*, the principal bacterium causing dental caries [18]. The powerful antibacterial property of QPEI was also demonstrated against various Gram-positive and Gram-negative microorganisms [15,18,19].

The present study evaluated the quaternary ammonium silica particle (QASi), which was incorporated into medical-grade silicone sheets. Samples of modified silicone were tested against microorganisms isolated from patients hospitalized at Meir Medical Center, Israel. Clinical isolates that are commonly associated with implantable medical devices, including *S. epidermidis*, *MSSA*, *MRSA*, and *E. faecalis*, were tested (all sensitive to amoxicillin/clavulanic acid, cefuroxime, and ofloxacin), as well as species of *P. aeruginosa* that are sensitive to ciprofloxacin. The modified silicone exhibited very potent antibacterial effects against all tested bacteria: starting with an inoculum of 10^6^ live bacteria, not a single bacterium survived at low QASi concentrations. Furthermore, we found no correlation between resistance/sensitivity to an antibiotic and sensitivity to QASi.

The modified silicone is unique, as the antibacterial components are not released into the surrounding milieu, as evident from the absence of an inhibitory halo around the material in the ADT. This unique property, which renders the novel QASi safe for use in human subjects, was tested and approved by the US FDA and purported to have superior non-decaying properties [16,17]. Antibacterial components released from medical devices might have adverse systemic or local biological effects. In addition, release from a device, which in some cases is intended for prolonged or even lifetime use, is disadvantageous, as slow and steady decomposition of the device and the antibacterial properties may occur over time.

With the temperature-controlled spectrophotometer and appropriate software, the DCT allows for the estimation of the number of viable bacteria at the end of the direct contact incubation period according to the calibration growth curves. In the DCT, bacteria cells contact the tested material under controlled conditions, and the growth of the surviving bacteria can be monitored and quantified.

The bactericidal mechanism of the immobilized quaternary ammonium silica was not fully characterized in this study, as it was previously characterized in other studies [12,18,19,30]. Over 50 years ago, several investigators suggested that quaternary ammonium compounds cause lysis of the bacterial cells by binding to the cell wall components, resulting in membrane destabilization and leakage of cytoplasm [30]. The QASi nanoparticles in the present study exhibited strong antibacterial effects against all the bacteria tested. The high antibacterial activity is likely due to a strong attraction of positively charged QASi to negatively charged bacterial cell surfaces. Lin et al. found that N-alkylated polyethyleneimine (PEI) was effective against various Gram-positive and Gram-negative bacteria [31,32]. From the limited number of bacteria tested in the current study and in earlier studies, it appears that the added QASi affected the Gram-positive bacterial growth to the same extent as that of the Gram-negative strain tested (*P. aeruginosa*). It is possible that after destroying the outer membrane’s permeable barrier, the cationic groups and the hydrophobic moiety further penetrate the inner membrane, causing leakage [33]. In contrast, Gram-positive bacteria such as *S. aureus*, *E. faecalis*, and *S. epidermidis* have a simple cell wall structure consisting only of a rigid peptidoglycan layer. Although thick, this layer has numerous pores that allow molecules to easily penetrate the cell wall and reach the cytoplasmic membrane [34]. Although it is not known how the initial damage to the outer and/or cytoplasmic membrane ultimately kills the bacteria, these nanoparticles presumably actively participate by inducing autolysis [35].

The current in vitro study is part of a larger series of studies testing the antimicrobial properties of QASi nanoparticles. Nanoparticles are advantageous due to their exceedingly large surface area relative to their size and scope. Thus, nanoparticles constructed from QASi, which contain an extremely high number of active antibacterial residues per particle, may provide strong activity, even though very few particles are added to the native matrix. The antibacterial properties of QASi led to a decrease in measured bacteria. This is a major goal of the tested material in terms of preventing the formation of biofilm, as once it forms, removal of the device is frequently inevitable.

## 5. Conclusions

The current study showed that incorporating low concentrations of QASi nanoparticles into medical-grade silicone resulted in no growth or in a significant decrease in all the microorganisms tested on them. This novel nanoparticle technology seems to be safe and effective, as demonstrated by the lack of diffusion into the aqueous surroundings. Incorporating nanoparticle technology into medical-grade silicone during production can potentially add an antimicrobial property to the medical device, thus inhibiting bacterial colonization and the onset of biofilm formation. While this novel technology shows great promise, additional strategies to reduce the contamination of implantable devices should be pursued. The safety of the present technology has been approved for use by the US FDA. The efficacy of incorporating this novel technology into silicone-grade medical devices merits further investigation in appropriate animal models before considering human studies.

## Data Availability

The raw and processed data that are required to reproduce these findings are available to download from. https://data.mendeley.com/preview/yh553s6gpt?a=0c66c209-25dc-49e8-a66e-00d1d515cf69 (accessed on 9 December 2024). Some of the processed data that are required to reproduce these findings cannot be shared at this time, as they are also part of an ongoing study.

## Abbreviations

ADT, agar diffusion test; BHI, brain–heart infusion; DCT, direct contact test; MRSA, methicillin-resistant *Staphylococcus aureus*; MSSA, *Methicillin-sensitive S. aureus*; OD, optical density; PEI, polyethyleneimine; QASi, quaternary ammonium silica; QPEI, quaternary ammonium cross-linked polyethyleneimine.
